# Formulation and Evaluation of Herbal-Based Antiacne Gel Preparations

**DOI:** 10.1155/2023/7838299

**Published:** 2023-12-18

**Authors:** Julia Afrakoma Ansong, Emmanuel Asante, Raphael Johnson, Mariam El Boakye-Gyasi, Noble Kuntworbe, Frederick William Akuffo Owusu, Kwabena Ofori-Kwakye

**Affiliations:** Department of Pharmaceutics, Faculty of Pharmacy and Pharmaceutical Sciences, College of Health Sciences, Kwame Nkrumah University of Science and Technology, Kumasi, Ghana

## Abstract

Acne vulgaris is an inflammatory skin condition that affects virtually everyone at some point. Papules, comedones, pustules, scarring, and nodules are standard features of the disease and can have a detrimental social and psychological impact on an individual. Although allopathic acne treatments are available, they have adverse side effects, are expensive, and are prone to cause antibiotic resistance. The present study is aimed at formulating and evaluating topical gels containing *Aloe vera*, *Allium cepa*, and *Eucalyptus globulus* extracts as potential antiacne drugs. Six formulations containing the herbal extracts were prepared using 1% Carbopol 940 as a gelling agent. The phytochemical composition of the plant extracts was determined. The extracts and gels' minimum inhibitory concentration (MIC) was assessed using the microbroth dilution method. The physicochemical properties of the formulated gels, such as homogeneity, colour, texture, odour, grittiness, spreadability, extrudability, viscosity, pH, and drug content, were evaluated. All the plant extracts contained alkaloids, flavonoids, tannins, triterpenoids, and coumarins. The gel formulations showed varying activity against *Staphylococcus epidermidis*, *Staphylococcus aureus*, *Escherichia coli*, *Candida albicans*, and *Pseudomonas aeruginosa* at various concentrations. The phytochemical components of the plant extracts are probably responsible for the antimicrobial activity of the gel formulations. The 5% *Aloe vera*-*Allium cepa* (1 : 1) combination gel formulation showed excellent activity against *Staphylococcus epidermidis*, *Staphylococcus aureus*, *Escherichia coli*, *Pseudomonas aeruginosa*, and *Candida albicans*, with MICs of 12.50, 25.00, 6.25, 25.00, and 12.50 mg/mL, respectively. The gels generally had good physicochemical and antimicrobial properties and could be used as antiacne remedies.

## 1. Introduction

Acne vulgaris, or acne, is a skin condition that affects many people and influences almost every individual [1]. It generally impacts individuals in their adolescence and young grown-ups and is stimulated by male chemicals brought about by the adrenal glands of both genders. It is, for the most part, observed on the face, chest, and back. Symptoms include pain, pustules (pimples), papules, tenderness, erythema, and loss of function [2].

Acne influenced around 650 million individuals worldwide in 2015 and was appraised as the eighth-most regular sickness universally [3–5]. Nationalities with hazier skin are more inclined to postprovocative hyperpigmentation, and it is more extreme in those with a positive family ancestry. Scars are known to lessen with age and time, yet this is very dubious [6]. Acne accounted for 5.3 percent of all skin diagnoses in a recent survey and is well-known as the second most common of all skin conditions [1, 7]. During puberty, it is more common in boys than girls; however, it is more prevalent in ladies than men during adulthood. Acne prevalence is related to diet [8, 9], weight, hereditary commitment, hormonal changes, and feelings of anxiety [10–12].

Pimples may happen when the sebaceous organs associated with pores liable for moving dead cells to the surface space of the skin get hindered. This blockade usually results in bacterial colonization and attack on the sebum, resulting in whiteheads, blackheads, and finally, inflammation and scars when the body's mechanism tries to fight back. *Propionibacterium acnes* and *Staphylococcus epidermidis* assume significant parts concerning inflammatory acne and shallow disease by utilizing sebaceous fatty substances into unsaturated fats, to which neutrophils are pulled in [13, 14].

Medicinal plants have the advantages of patient tolerance and wide acceptability [15]. For example, *Azadirachta indica* and *Citrus aurantifolia* have been folklorically used as antiacne agents [16–18]. *Aloe vera*, *Allium cepa*, and *Eucalyptus globulus* have antimicrobial, anti-inflammatory, and antioxidant activities that significantly treat skin infections. They are additionally known to have nutrients and minerals to improve the general strength of the skin. Acne accounted for 5.3% of all skin diagnoses reported, and acne vulgaris was the second most common by gender [1, 19, 20].

In Ghana, only some acne measures are available since many individuals get their acne treated at their local pharmacy and buy over-the-counter acne treatments, particularly topical skincare medications. As a result, there needs to be more data on acne. The method of acne treatment is gradually shifting away from allopathic prescriptions, which are expensive when considering the total cost of treatment and are also known to elicit bacterial resistance [15]. People who suffer from acne want quick remedies to boost their self-esteem. This study is aimed at formulating and evaluating the effectiveness of an active antiacne gel against *P. aeruginos*a and *Staph. epidermidis* with excellent synergistic effect against other microbial strains [17, 21–26].

## 2. Materials and Methods

### 2.1. Materials

*Eucalyptus globulus* (family: Myrtaceae) oil, *Aloe vera* (family: Liliaceae) leaves, and *Allium cepa* (family: Liliaceae) bulbs (*Allium cepa*) were purchased from Adum, Kumasi, Ghana. The plant materials were authenticated at the Department of Herbal Medicine, KNUST, Kumasi, Ghana, by Mr Clifford Asare. *Staphylococcus epidermidis* (clinical strain), *Staphylococcus aureus* (ATCC 25923), *Escherichia coli* (ATCC 25922), *Pseudomonas aeruginosa* (ATCC 4853), and *Candida albicans* (clinical strain) were obtained as pure isolate cultures (KCCR, KNUST) and used as test microorganisms. The Mueller-Hinton broth (Oxoid Ltd., Basingstoke, UK), 0.5 McFarland (Hardy Diagnostics), diethyl ether, ethanol, and distilled water were obtained from the Department of Pharmaceutics, KNUST, Ghana.

### 2.2. Methods

#### 2.2.1. Preparation of *Aloe vera* and *Allium cepa* Extracts

*(1) Aloe vera Extract*. The matured leaves were washed with diethyl ether and distilled water. The gel was drained when the parenchymatous layer of the leaves was peeled away. A mortar and pestle were used to create the slurry. After that, the new gel was drained, and the weight was recorded. The gel was utilized in the formulation [27]. This procedure was carried out in triplicate.

*(2) Allium cepa Extract*. About 165 bulbs of *Allium cepa* were peeled, washed, crushed, and homogenized into smaller pieces and air-dried at 26 ± 1°C. About 637.61 g of the pulverized *Allium cepa* was weighed and soaked in 4 L of ethanol for 168 hours, filtered, and evaporated to dryness using the water bath to obtain a concentrate [21, 28]. The extractive procedure was repeated, and the weights of the concentrates were recorded.

#### 2.2.2. Identification and Chemical Composition of *Eucalyptus globulus* Oil

The volatile oil of *Eucalyptus globulus* was stored at room temperature in an amber-coloured bottle to avoid rancidity. An identification and chemical composition test was performed on the oil to ascertain its quality and chemical constituents using gas-liquid chromatography [23, 29, 30].

#### 2.2.3. Determination of Percentage Yield

An analytical balance (model Kern PCB 1000-2) was used to determine the total weight of raw material before extraction and the weight of extract after extraction. From these extracts, the percentage yield was calculated using the following formula:

(1) $$\begin{array}{c} \%\text{yield}=\frac{\text{Weight of extract}}{\text{weight of total raw material}}\times 100 \end{array}$$

#### 2.2.4. Phytochemical Analysis

Phytochemical screening was performed to determine the secondary metabolites in the various plant materials. Using previously established protocols, the secondary metabolites investigated were tannins, alkaloids, flavonoids, triterpenoids, sterols, glycosides, coumarins, and saponins [27, 31, 32].

#### 2.2.5. Preparation of Mueller-Hinton Broth (Single and Double Strength)

For single strength, 2.1 g Mueller-Hinton broth was dissolved in 100 mL sterile water, transferred to 10 mL test tubes, and sterilized for 30 minutes in an autoclave at 121°C [33]. For double-strength, 4.2 g was weighed and dissolved in 100 mL sterile water, transferred into test tubes of 10 mL each, and sterilized in an autoclave at 121°C for 30 minutes, cooled, and stored at room temperature; 25°C.

#### 2.2.6. Subculture of Microorganisms

*Staphylococcus epidermidis* and *Staphylococcus aureus* (gram-positive), *Escherichia coli* and *Pseudomonas aeruginosa* (gram-negative), and *Candida albicans* (fungi) are the organisms used. The Mueller-Hinton broth test tube was sterilized before use by flaming the mouth with a Bunsen burner under a laminar flow cabinet. Then, 1 mL of the pure isolate was introduced into the broth, flamed again, and capped with a cork to be incubated at 37°C for 24 hours [27, 33].

*(1) Streaking of the Subcultured Organisms to Obtain Pure Isolates*. The agar was prepared, transferred into test tubes, and sterilized in an autoclave for 30 minutes at 121°C. It was poured onto a petri dish and cooled in the safety cabinet. The subcultured organisms were streaked over the surface of the agar and incubated for 24 hours with a sterile inoculum loop [27, 33].

#### 2.2.7. Estimation of Minimum Inhibitory Concentrations (MIC) of Plant Materials

The microbroth dilution technique was employed to estimate the MIC. The microtiter plate was filled with appropriate and calculated amounts of the Mueller-Hinton nutritious broth, sterile water, distinct plant materials concentrations, and microorganisms (as compared to the McFarland standard) and incubated at 37°C for 24 hours. The MIC was determined using test formulations with the lowest dilution concentration and no apparent growth [27, 34–38].

#### 2.2.8. Formulation of Individual Gels

Gels of the samples (Eucalyptus and *Allium cepa*) and aloe gel were prepared at a concentration of 5% after preformulation trials (Table 1). Carbopol 940 was placed in distilled water in a separate beaker with constant stirring. Propylparaben and methylparaben were dissolved in 5 mL of distilled water in another beaker. The extracts were added to the solution and thoroughly levigated. Afterward, the combination above was added to the carbopol mixture and mixed well. Finally, with steady and continuous stirring, propylene glycol and triethanolamine were added to the dispersion in drops, and the pH was adjusted to 6.8-7.4 (neutralized) [17, 39].

#### 2.2.9. Formulation of Combination Gels

Combination gels were prepared at the same concentration of 5%, which corresponded to 10 times the minimum inhibitory concentration values. Carbopol 940 was distributed uniformly in a separate beaker in water with continuous stirring. Propylparaben and methylparaben were dissolved in 5 mL of water in another beaker. To the solution, the extracts were added and levigated well. The above mixture was then added to the carbopol mixture and stirred well. Finally, propylene glycol and triethanolamine were added in drops to the dispersion with constant and continuous stirring, and also the pH was adjusted to 6.8-7.4 (neutralized) [17].

#### 2.2.10. Physicochemical Evaluation of Individual and Combination Gels

Official techniques were used to assess pH, colour, odour, consistency, texture, greasiness, homogeneity, grittiness, compatibility, drug content, viscosity, extrudability, and spreadability [17, 40–43].

#### 2.2.11. Estimation of MIC of Formulated Gels

The microbroth dilution technique was employed to estimate the MIC. The microtiter plate was filled with appropriate and calculated amounts of the Mueller-Hinton nutritious broth, sterile water, distinct concentrations of the formed gels, and microorganisms (compared to the McFarland standard) and incubated at 37°C for 24 hours. Three wells of the microtiter plate were used for each test organism. A test tube with extract/oil was used as a positive control, and no organism was present in the negative control. The MIC was determined using test formulations with the lowest dilution concentration and no apparent growth [27, 34–38].

## 3. Results and Discussion

### 3.1. Identification and Chemical Composition of Eucalyptus Oil

**Figure 1:**
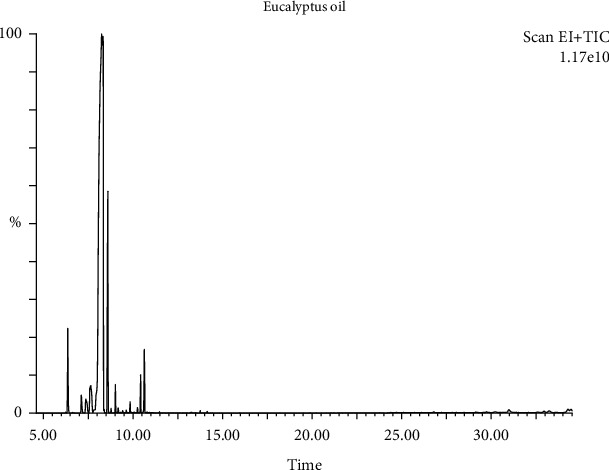
Identification test on eucalyptus oil using GC-MS.

The chemical composition of *Eucalyptus globulus* oil was determined using gas chromatography-mass spectroscopy. Eucalyptus plants produce terpenoid hydrocarbons and essential oils (eucalyptus oils), and they can be categorized as medicinal, industrial, or perfumery, depending on their chemical makeup. The eucalyptus product's GC/MS total ion chromatogram was created under the conditions described above, and the results are presented in Figure 1. The softcopy findings of GC/MS Turbo Mass utilizing peak area normalization measurements were used to determine the concentration of all the detected chemicals based on peak area/peak height. The two major detected peaks and chemical constituents were those of 1, 8-cineole, and terpinene (Table 2). Eucalyptus oil is deemed therapeutic if it contains not less than 70% of 1, 8-cineole [44–46].

The eucalyptus oil studied contained more significant than 70% of 1,8-cineole, suggesting that the oil may be utilized for therapeutic purposes [46, 47]. Genetic factors determine the chemical composition of essential oils. However, other variables, including location, the vegetative cycle, the method of production, and environmental elements, including soil characteristics, relative humidity, solar radiation, temperature, and hydric stress, can significantly alter chemical constituent production [46, 48].

### 3.2. Phytochemical Composition of Plant Materials

Phytochemicals have pharmacological properties that can treat several ailments, including bacterial and fungal infections. The bioactive chemicals saponins, glycosides, flavonoids, alkaloids, and tannins were discovered due to the phytochemical screening (Table 3). Secondary metabolites found in plant materials have been shown to have antimicrobial activity against pathogenic bacteria. Phenolic compounds (tannins) and alkaloids are known to inhibit the activities of pathogenic organisms and therefore are responsible for the inhibitory activities observed by the gel. [27, 31, 32].

Plant-derived biologically active molecules are often considered more acceptable and less hazardous than synthetic substances, providing many potential disease-controlling drugs. Secondary metabolites can control pathogenic organisms, overcoming the problems associated with produced chemicals, according to research into plant biochemistry, physiology, and natural product chemistry. As a result, there is rising interest in developing alternative microbial contamination control strategies that reduce or eliminate the need for antibiotics [27, 31, 32].

### 3.3. Microbiological Evaluation of Extracts and Formulated Gels

The MIC of the extracts against the tested organisms is required to calculate the dose in the formula. This MIC was then increased by ten to generate the dose, which was then utilized to calculate the amount of extract used in the gel formulations. The MIC results are shown in Tables 4 and 5. The antibacterial properties of the plant extracts were tested using the microbroth dilution technique. The findings of this study showed that the plant extracts were active against the test organisms.

The plant materials exerted antimicrobial action at different concentrations against the organisms tested. *Allium cepa* extract showed activity against all the test organisms. *Eucalyptus globulus* oil and *Aloe vera* gel also exhibited activity against all the organisms except *Pseudomonas aeruginosa* (*Aloe vera* and eucalyptus oil) and *E. coli* (*Aloe vera*) (Table 4), indicating that products derived from the plant extracts could be beneficial in treating infections caused by these microorganisms. *Allium cepa* juice was not used in the gel formulation because of its weak activity compared to the ethanolic extract. Crude extract activity is deemed significant if MIC values are less than 100 *μ*g/mL, moderate when 100 *μ*g/mL to 625 *μ*g/mL, or low when 625 *μ*g/mL or above [49, 50]. Based on this information, the extracts and oil possess low activity.

The different gel formulations showed inhibitory effects against *Staphylococcus epidermidis* and *Staphylococcus aureus* at 6.25, 12.5, and 25 mg/mL (Table 5). Only formulations containing *Aloe vera* only and eucalyptus oil exhibited the most antibacterial effect against *Staph epidermidis*. This finding is comparable to previous studies [44, 51, 52]. Nonetheless, *Allium cepa* only formulation was most active against *Staph aureus*. This result may explain why the three plant extracts treat many diseases [25].

*Pseudomonas aeruginosa* has been linked to skin infections, especially at exposed sites like wounds and pressure sores, and can also affect burst and infected nodules and papules. In this current study, *Allium cepa* only and *Allium cepa* in combination with *Aloe vera* were the most active against *Pseudomonas* with an inhibitory value of 25 mg/mL. *Aloe vera* alone was not active against *Pseudomonas*, as reported by Goudarzi et al. [53]. The inhibitory action of these gels against *P. aeruginosa* explains their potential advantage in treating skin infections with such organisms implicated.

The gel formulations exerted broad antimicrobial action against gram-positive and gram-negative bacteria and the fungus. The growth of *Candida albicans* was likewise suppressed most by the *Allium cepa*, *Aloe vera*, and *Eucalyptus* combination gel with an inhibitory value of 6.25 mg/mL. *Aloe vera* having inhibition against *Candida albicans* was similar to that reported by Stanley et al. [25]. There are several clinical manifestations of candidiasis, but most commonly involve mucosa surfaces and deep-seated infections. This study contradicts [54], who reported that *Aloe vera* gel had no inhibitory effects against *Candida albicans*. All gel formulations inhibited *Escherichia coli* with different MICs of 3.125, 6.25, and 25 mg/mL except the *Aloe vera* gel formulation, which contradicts the findings of Stanley et al. [25], who observed inhibition in their study. *Allium cepa*-only gel formulation showed the most significant inhibition. Eucalyptus oil was active against *E. coli* (gram-negative organism), which aligns with previous findings [48, 50, 53].

The antimicrobial property of *Allium cepa*-*Aloe vera* combination gel had a four (4) fold increase in activity against *S.aureus* compared to *Aloe vera* gel and a two (2) fold activity against *E.coli.* The increased antimicrobial activity of the *Allium cepa*-*Aloe vera* combination gel may be due to the synergistic activity of the *Allium cepa* and *Aloe vera* gels (Table 5). *Allium cepa*-*Eucalyptus* gel combinations had a four (4) fold increase in antimicrobial activity against *S.epidermidis* compared to *Eucalyptus* gel alone (Table 5). Table 5 further revealed that some formulations were bacteriostatic while others were bactericidal on the test organisms. The current research reveals the formulations' therapeutic potential in antibacterial and antioxidant properties. According to the results of the tests mentioned above, the extracts have a significant growth inhibitory action on the organisms. The efficacy of these formulations based on MIC values supports their use in the prevention and treatment of bacterial infections caused by a variety of pathogenic bacteria with antibiotic resistance, most importantly, acne [55].

### 3.4. Quality Control Tests on Formulated Topical Gels

#### 3.4.1. Organoleptic Characteristics of the Gels

Physical appearance, pH, colour, odour, consistency, greasiness, grittiness, homogeneity, texture, compatibility, viscosity, spreadability, drug content, and extrudability were examined for the six gel formulations. The study's findings were within the ICH standards' allowed limits, and the specifics are reported in Tables 6 and 7. The produced gels were homogeneous and uniform in appearance, odour, and consistency. All formulations had pH values close to neutral (6.80-7.49), indicating they may not cause skin irritation. The gel formulations were visually examined for colour using a colour codebook. The produced formulations were transparent without the active components in the preformulation studies but exhibited different colours in the formulation studies (Table 7). All the developed gel formulations were homogeneous and free of lumps.

#### 3.4.2. Physicochemical Properties of Carbopol (Gelling Agent) and Formulated Gels

The spreadability of gels is crucial because it shows how the gel acts once removed from the tube. According to the spreadability parameters (Table 7) [42], the gel formulations are easily spreadable and can be easily applied to the skin after removal from the primary packaging material. Also, more than 90% of the contents of most of the gel formulations were extrudable, indicating excellent extrudability. The gel can, therefore, be easily removed from its primary package with minimal stress/force. A few had greater than 80% extrudability (Table 7) (extrudability: >90%: excellent; >80%: acceptable; >70%: fair) [40]. The viscosity of gels is known to impart the spreadability and extrudability of gels. Therefore, the viscosity of formulated gels should be characterized and ensured that they produce gels with optimum spread and extrudability [39, 40]. The spreadability and extrudability results corroborate that the formulated gels had suitable viscosities (Table 7). The percentage of active plant content also fell within the range of 90.68 to 99.83% and conformed to the USP limit for drug content. The values indicate that the gels contained uniform amounts of active herbal constituents (Table 7) and will be able to deliver the expected amount of active constituents to the site of action [42, 56].

## 4. Conclusion

Acne vulgaris is a reasonably prevalent condition that affects virtually everyone at some point. Herbal medications are considered safer than allopathic drugs; therefore, the current formulations can be recommended as an effective tool for managing and treating acne. According to the findings from this study, varying combinations of *Aloe vera* leaf extracts, *Eucalyptus globulus* oil, and *Allium cepa* bulb extracts have effective synergistic therapeutic characteristics for managing acne vulgaris. The 5% *Aloe vera*-*Allium cepa* (1 : 1) combination gel was most active against *Staphylococcus aureus*, *Escherichia coli*, *Pseudomonas aeruginosa*, and *Candida albicans* and possessed the requisite physicochemical properties for use in acne vulgaris.

## Figures and Tables

**Table 1:** Constituents for the formulation of gels.

Ingredient	Part used	Concentration (%)	Use/function
Ethanolic extract of *Allium cepa*	Bulb	5	Antiscarring, anti-inflammatory
A slurry of *Aloe vera*	Large leaves	5	Kills acne-causing bacteria, scars, moisturizer
Extract of *Eucalyptus globulus* by hydro distillation	Leaves	5	Antiacne agent
Carbopol 940	—	1	Gelling agent
Methylparaben	—	0.1	Preservative
Propylparaben	—	0.1	Preservative
Triethanolamine	—	2	Neutralizer
Propylene glycol	—	2	Humectant
Distilled water	—	QS	Vehicle

**Table 2:** Chemical composition of eucalyptus oil determined by GC-MS.

No.	Chemical compound	Chemical content (%)
1.	1,8-Cineole	78.13-78.35
2.	Terpinene	7.30-9.35
3.	Pinocarveol	0.26-0.34
4.	*α*-Terpineol	1.98-2.53
5.	Terpinyl acetate	0.60-0.76
6.	Terpinene-4-ol	0.89-1.14
7.	Linoleic acid	0.43-0.55
8.	*α*-Pinene	2.34-2.98
9.	*β*-Pinene	0.71-0.91
10.	Cyclycloeucalenol	0.19-0.24
11.	*α*-Myrcene	1.09-1.40
12.	*α*-Amyrin	0.38-0.48
13.	Minolinolenin	0.22-0.28
14.	Squalene	0.35-0.45

**Table 3:** Phytochemical constituents of plant materials.

Extract	Tan	Gly	Sap	Flav	Alka	Trit	Ste	Coum
^∗^Eucalyptus	+	+	+	+	+	+	-	+
*Allium cepa*	+	+	+	+	+	+	+	+
Aloe	+	-	-	+	+	+	-	+

Key: absent (-); present (+); Tan = tannins; Gly = glycosides; Sap = saponnins; Flav = flavonoids; Alka = alkaloids; Trit = triterpenoids; Ste = sterols; Coum = coumarins; ^∗^oil.

**Table 4:** Mean MICs (mg/mL) of the different plant materials against test organisms.

Plant materials	*Staphylococcus epidermidis*	*Staphylococcus aureus*	*Escherichia coli*	*Pseudomonas aeruginosa*	*Candida albicans*
Aloe gel	0.625 ± 0.003	2.500 ± 0.040	-	-	1.250 ± 0.005
Eucalyptus oil	0.625 ± 0.005	2.500 ± 0.050	2.500 ± 0.060	-	1.250 ± 0.020
Allium extract	0.156 ± 0.002	0.625 ± 0.010	0.313 ± 0.002	2.500 ± 0.007	1.250 ± 0.030
Allium juice	2.500 ± 0.050	10.000 ± 0.030	40.000 ± 0.100	-	10.000 ± 0.009

Key: (-) no inhibition.

**Table 5:** Mean MICs (mg/mL) of the different gel formulations against test organisms.

Gel formulations	*Staphylococcus epidermidis*	*Staphylococcus aureus*	*Escherichia coli*	*Pseudomonas aeruginosa*	*Candida albicans*
5% *Aloe vera* gel	6.25 ± 0.02	25.00 ± 0.05	-	-	12.50 ± 0.11
5% *Eucalyptus globulus* gel	6.25 ± 0.06	25.00 ± 0.20	25.00 ± 0.13	-	12.50 ± 0.21
5% *Allium cepa* gel	12.50 ± 0.07	6.25 ± 0.31	3.13 ± 0.15	25.00 ± 0.22	12.50 ± 0.22
5% *Allium cepa* and *Aloe vera* gel (1 : 1)	12.50 ± 0.10	25.00 ± 0.22	6.25 ± 0.21	25.00 ± 0.10	12.50 ± 0.08
5% *Allium cepa* and *Eucalyptus* gel (1 : 1)	25.00 ± 0.12	12.50 ± 0.12	25.00 ± 0.50	-	25.00 ± 0.35
5% *Allium cepa*, *Eucalyptus*, and *Aloe vera* gel (1 : 1 : 1)	25.00 ± 0.13	25.00 ± 0.08	25.00 ± 0.31	-	6.25 ± 0.43

Key: (-) no inhibition.

**Table 6:** Organoleptic characteristics of carbopol (gelling agent) and formulated gels.

Gel	Conc (% w/v)	Colour	Odour	Consistency	Greasiness	Grittiness	Homogeneity	pH	Texture	Patient acceptability
Carbopol	1	Colourless	Characteristic	Very consistent	Nongreasy	Nongritty	No lump	6.81 ± 0.11	Smooth	Acceptable
*Allium cepa*	5	#FEB475 (soft orange)	Characteristic odour	Consistent	Nongreasy	Nongritty	No lump	7.16 ± 0.30	Smooth	Acceptable
*Eucalyptus*	5	255,255,255 (white)	Minty	Consistent	Nongreasy	Nongritty	No lump	7.35 ± 0.21	Smooth	Acceptable
Aloe	5	A8003-0 (clear)	Characteristic odour	Consistent	Nongreasy	Nongritty	No lump	7.15 ± 0.10	Smooth	Acceptable
*Allium cepa* and *Eucalyptus*	5 (1 : 1)	#FCDE9C (very soft orange)	Characteristic minty odour	Consistent	Nongreasy	Nongritty	No lump	7.18 ± 0.08	Smooth	Acceptable
*Allium cepa* and Aloe	5 (1 : 1)	#EEB479 (soft orange)	Characteristic odour	Consistent	Nongreasy	Nongritty	No lump	6.85 ± 0.13	Smooth	Acceptable
*Allium cepa*, Aloe, and *Eucalyptus*	5 (1 : 1 : 1)	#FFF3B2 (pale yellow)	Characteristic minty odour	Consistent	Nongreasy	Nongritty	No lump	7.43 ± 0.11	Smooth	Acceptable

The numbers in parentheses are ratios of the plant materials used.

**Table 7:** Physicochemical properties of carbopol (gelling agent) and formulated gels.

Gel formulation	Concentration (% w/v)	Spreadability (50 g) (cm)	Extrudability (%)	Viscosity (Cp) (speed 6)	Active plant content (%)
Carbopol	1	0.9 ± 0.006	94.75 ± 0.026	795.0 ± 0.653	—
*Allium cepa*	5	1.2 ± 0.017	95.60 ± 0.065	140.0 ± 0.364	99.83 ± 0.020
*Eucalyptus*	5	0.7 ± 0.033	80.70 ± 0.392	630.0 ± 0566	97.91 ± 0.006
Aloe	5	1.1 ± 0.011	93.45 ± 0.041	306.7 ± 0.173	99.29 ± 0.053
*Allium cepa and Eucalyptus* (1 : 1)	5	1.3 ± 0.011	84.00 ± 0.065	216.7 ± 0.493	90.68 ± 0.052
*Allium cepa* and Aloe (1 : 1)	5	1.4 ± 0.007	95.35 ± 0.041	125.0 ± 0.000	99.32 ± 0.131
*Allium cepa*, *Eucalyptus*, and Aloe (1:1 : 1)	5	1.2 ± 0.017	87.95 ± 0.043	148.3 ± 0.196	99.52 ± 0.011

## Data Availability

The data used to support the findings of this study are included in the article.
